# Dosimetric evaluation of GAFCHROMIC® XR type T and XR type R films

**DOI:** 10.1120/jacmp.v6i1.2051

**Published:** 2005-03-17

**Authors:** Sharifeh A. Dini, Rafiq A. Koona, John R. Ashburn, Ali S. Meigooni

**Affiliations:** ^1^ Department of Radiation Medicine University of Kentucky Medical Center 800 Rose Street Lexington Kentucky 40536 U.S.A.

**Keywords:** dosimetry, GAFCHROMIC® film, XR type T film, XR type R film

## Abstract

The high spatial resolution of radiochromic film makes it ideal for dosimetric measurements and dose distributions in regions of high dose gradient. Intensity‐modulated radiation therapy, intravascular brachytherapy, and eye‐plaque radiation therapy demand precise spatial dosimetric calculations. Such precision is not possible with conventional dosimeters, such as thermoluminescent dosimeters and ionization chambers. Recently, new GAFCHROMIC® XR type T and type R films have been developed for radiation dosimetry, specifically in interventional radiology procedures. Dosimetric characteristics (i.e., linearity, post‐exposure density growth, energy dependence, dose‐rate dependence, and UV light sensitivity) of these new films were investigated. To evaluate the clinical applications of these films, their characteristics were compared with other commercially available film models. GAFCHROMIC® XR type T and type R films were found to be more sensitive to low‐energy doses as compared with GAFCHROMIC® MD‐55 films.

PACS numbers: 87.66‐a, 87.53‐j

## I. INTRODUCTION

Dosimeters with high spatial resolution are desirable for measurement and quality control of radiation in high‐dose gradient areas. These detectors could provide accurate dose distribution in the close vicinity of brachytherapy sources. Two procedures in particular that require higher resolution dosimeters are intravascular brachytherapy,^(1)^ ^,^ ^(2)^ for the treatment of restenosis, and eye‐plaque radiation therapy,^(3)^ for the treatment of ocular melanoma. Moreover, the quality assurance of the intensity‐modulated radiation therapy technique could also benefit from these types of detectors.^(^ ^4^ ^–^ ^7^ ^)^ Conventional measuring systems such as thermoluminescent dosimeters, ionization chambers, and semiconductors are not capable of such spatial resolution. Although radiographic silver halide film provides high spatial resolution, it is not a tissue‐equivalent detector due to its high *z*‐component. Moreover, the high *z*‐element of this film type increases its energy dependence, particularly at low‐energy radiation levels. Conversely, the organic‐based radiochromic film dosimeters offer superior tissue‐equivalent properties and high spatial resolution that could fulfill the dosimetric requirements of the latest advancements in the field of radiation therapy.

GAFCHROMIC®, or radiochromic, films were originally designed for industrial applications by International Specialty Products (ISP), Inc. (Wayne, NJ). In radiochromic films an organic‐based dye is used, which changes color due to polymerization when exposed to radiation. Various superior features of this film compared with silver halide film include the following: relatively low‐energy response, ^(8)^ insensitivity to visible light, self‐developing characteristics, and dose‐rate independence.^(9)^ These features have attracted the attention of medical physicists for the use of GAFCHROMIC® films in medical applications.^(10)^ Detailed studies were performed by McLaughlin et al.^(^ ^11^ ^–^ ^13^ ^)^ to determine the dosimetric properties of the original models of radiochromic films. The recent improvements in film sensitivity, accuracy, precision of film manufacturing, and ease of use have further extended the medical applications of these film types for different treatment modalities, such as brachytherapy,^(8)^ ^,^ ^(14)^ ^,^ ^(15)^ interface studies,^(16)^ ^,^ ^(17)^ stereotactic radio surgery,^(^ ^13^ ^,^ ^18^ ^–^ ^20^ ^)^ dosimetry in the penumbra region,^(21)^ and proton dosimetry.^(22)^ Butson et al. have extensively investigated the effects of polarization and light sources on the response of MD‐55‐2^(23)^ and HS^(^ ^24^ ^–^ ^26^ ^)^ GAFCHROMIC® media. Applications of these films have been discussed in detail in AAPM Task Group 55 (TG‐55).^(27)^

Recently, ISP developed GAFCHROMIC® XR type R and type T films for the measurement of high radiation dose in interventional radiological procedures. This film is very useful for direct measurements of skin dose from fluoroscopic procedures. The sensitivity of these films to photons with energies less than 200 keV is substantially greater than HD‐810 and MD‐55 films. Thomas et al. discussed various means of measuring the response of the XR type R films using different commercially available densitometers and flatbed scanners.^(28)^ The characterization of the XR type T films was presented as an abstract by Haque et al.^(29)^ in the World Congress on Medical Physics and Biomedical Engineering in 2002. Also, characterization of the HS and XR type R film was presented as an abstract by Ashburn et al.^(30)^ in the 2001 annual meeting of the American Association of Physicists in Medicine (AAPM). However, to our knowledge there is no publication to date on the dosimetric characteristics of the XR type T and type R media.

The main objective of this project was to determine the dosimetric characteristics (sensitivity, energy dependence, linearity, post‐exposure density growth, dose‐rate dependence, and UV light sensitivity) of the XR type T and type R films. This report offers a complete dosimetric evaluation of XR type T and type R films, along with a comparison of these films with commonly available film models for both low‐ and high‐energy X‐ray beams.

## II. MATERIALS AND METHOD

### A. GAFCHROMIC® XR type T and type R films

Two different types of GAFCHROMIC® XR films—type T and type R—were studied in these investigations. In both film types, the active layer is sandwiched between the two sheets of polyester. In the type T film, both polyester substrates are transparent, whereas in type R one is transparent and the other is opaque. These film types are designed and manufactured by ISP and are available in sheet sizes of 14in.×17in. (type R) and 5in.×5in. (type T). The color of XR type R film turns from amber to dark greenish‐black, and XR type T films turn from orange to brownish‐black, depending on the level of exposure.

### B. Dosimetric evaluation

The dosimetric characteristics of the XR type T and type R films were determined by cutting sheets of these films into small 4 cm×4 cm squares. These pieces were exposed to low‐energy X‐ray beams ranging from 10 kVp to 150 kVp from an Oldeft Therapax HF 150T superficial unit (Oldeft/Nucletron, Columbia, MD), and high‐energy X‐ray beams of 6 MV and 18 MV from a Varian LINAC (Clinac 2100C/D, Varian Oncology Systems, Palo Alto, CA). The effective energies and half value layers of the beams from a superficial unit are shown in Table 1.

**Table 1:** Nominal energies, half‐value layers, and effective energies of X‐rays and gamma rays used in these investigations

Nominal energy	Half‐value layer (mm A1)	effective energy (keV)
20 kVp	0.12	11
30 kVp	0.35	16
40 kVp	0.62	19
50 kVp	0.47	18
80 kVp	1.2	25
80 kVp	2.2	30
100 kVp	4.8	41
120 kVp	6.9	50
150 kVp	12.5	79
^99m^Tc	—	140
Ir192	—	380
6 MV	—	2000
18 MV	—	6000

The low‐energy measurements were performed in air by placing the film on an empty box with a 14 cm×14 cm cutout on its top. A layer of clear transparency was taped on top of the cutout area in order to support the film. Another transparency was placed on the film to hold the film in place and to provide a similar setup used for the calibration of the superficial unit using the Markus chamber (PTW‐New York, Hicksville, NY). All the low‐energy exposures were performed using a 15‐cm diameter cone. For high‐energy exposures, an appropriate buildup material of Solid Water™ phantom (Radiation Measurements Inc., Middleton, WI) was used.

The radiochromic films used were XR type T (lot number 30189‐1B), XR type R (lot number 30198‐2), and MD 55‐2 (lot number 37175). These films were handled in accordance with the precautions recommended in TG‐55.^(27)^

The XR type T films were scanned before and after exposure with a LUMISCAN‐50 laser film scanner (Lumisys, Sunnyvale, CA) using a spatial resolution of 0.35 mm. The optical densities of these films along the two orthogonal axes were corrected for background (reading from pre‐exposed film) and averaged for each piece of film using the PTW software Mephysto v6.30 (PTW‐New York, Hicksville, NY). Optical densities of XR type R films were read by a GretagMacbeth D19C reflection densitometer (GretagMacbeth, New Windsor, NY). This densitometer provides readings in four different colors: black, yellow, cyan, and magenta. While ISP recommends using cyan for this densitometer, the other colors were also studied in these investigations. The optical densities taken from five different points on the film, using the center and one point from each quadrant, were averaged and corrected for background to obtain the net optical density. This procedure was followed throughout these investigations. The MD‐55‐2 films were read with a GretagMacbeth D19C reflection densitometer for comparisons with XR type R films. A white paper was used as a backing material for reading the MD‐55‐2 films with this densitometer. For comparison of XR type T with MD‐55‐2 films, a LUMISCAN laser scanner was used to scan both film types. To minimize the UV exposure, these films were kept in black envelopes when not in use and were also stored in normal room temperature and humidity conditions.^(^ ^11^ ^,^ ^31^ ^–^ ^33^ ^)^ The measured optical densities were used to determine the dosimetric characteristics of the XR type T and type R films as described in the following sections.

### B.1 Film sensitivity

The sensitivities (i.e., response per unit absorbed dose) of XR type T, type R films, and MD‐55‐2 films were measured and compared in this study. The MD‐55‐2 data was also obtained along with XR type T and type R films in order to have an identical experimental setup for comparisons of these film types. This process eliminates any uncertainties that may arise when comparing the new films with the published data for MD‐55‐2 films measured with different scanning techniques. Four pieces of each film (XR‐type T and MD‐55‐2 films) were exposed to a 100‐kVp beam, and four more pieces of each film were exposed to a 6‐MV beam for the doses ranging from 186 cGy to 1856 cGy. These films were scanned with the LUMISCAN‐50 laser film scanner after 24 h, and their results were analyzed to obtain the film sensitivity. Similarly, four pieces of each film (XR‐type R and MD‐55‐2 films) were exposed to a 50‐kVp X‐ray beam for the following doses: 184 cGy, 460 cGy, 920 cGy, and 1840 cGy. After 24 h, these films were read with the GretagMacbeth D19C reflection densitometer. A white paper was used as a backing material for reading the MD‐55‐2 films with this densitometer. The sensitivity of each film type for different beam qualities was determined by measuring the optical density of the films as a function of absorbed dose. The sensitivities of different film types were compared as a function of scanning techniques.

### B.2 Energy dependence

The energy dependence of the XR type T film was evaluated by measuring the optical densities of 13 pieces of film exposed to 800 cGy, with different beam energies ranging from 20 kVp to 18 MV. A dose of 800 cGy was selected to optimize the signal‐to‐noise ratio and improve the accuracy of the data. One piece of film was exposed to a 6‐MV X‐ray beam and another to an 18‐MV X‐ray beam from a LINAC. Nine other pieces of film were exposed to low‐energy beams ranging from 11 keV to 79 keV (i.e., 20 kVp to 150 kVp) from a superficial unit. One piece of film was exposed to the Ir192 HDR (high dose rate) source (VariSource, Varian Oncology Systems, Palo Alto, CA) with an average energy of 380 keV, and another piece of film was exposed to ${\text{Tc}}^{99m}$ (Cardinal Health, Lexington, KY) with an average energy of 140 keV. For Ir192 HDR source irradiation, the film was placed on a 10cm×20cm×20cm piece of Solid Water™ phantom material. A Styrofoam block with a circular cutout at the center was used to hold the Ir192 source, in a single dwell position, using an endobronchial catheter. The film was kept at a distance of 4.8 cm directly beneath the HDR source. The irradiation with ^99m^Tc was performed by placing a vial containing 438 mCi of ${}^{99m}\text{Tc}$ on the film, which was located on the surface of a 10‐cm thick Solid Water™ phantom material. The initial dose rate of this vial was measured using an NIST calibrated Markus ionization chamber prior to the film exposure. Both the calibration and experiment were performed with identical geometric setups. The exposure time was adjusted to correct for the decay of the source during the irradiation. These films were scanned after 24 h with the LUMISCAN laser scanner. The variation of XR type T film response as a function of beam energy was used to evaluate the energy dependence of the film response. In addition, three pieces of MD‐55‐2 film were exposed to a fixed total dose of 800 cGy with different beam energies of 50 kVp, 100 kVp, and 6‐MV X‐ray beams. These films were read with the LUMISCAN laser scanner after 24 h, and the results were compared with those of XR type T films.

Similarly, 13 pieces of XR type R film and 6 pieces of MD‐55‐2 film were exposed to 400 cGy with energy beams in the range 11 keV to 6000 keV. The XR type R film is more sensitive, so a dose of 400 cGy was found sufficient to optimize the signal‐to‐noise ratio and hence to improve the accuracy of the experimental data. These films were read with the GretagMacbeth D19C reflection densitometer 24 h after the time of exposure. The responses of XR type R and MD‐55‐2 films for four different color spectra of the densitometer were used to determine the energy dependence of these film types.

### B.3 Linearity of the films

The linearity of XR type T film response was examined by exposing seven pieces of film to doses ranging from 46 cGy to 1856 cGy with a 100‐kVp X‐ray beam, and another eight pieces were exposed to doses ranging from 10 cGy to 3000 cGy with a 6‐MV X‐ray beam. These films were then scanned 24 h after the time of exposure using the LUMISCAN‐50 laser film scanner. Variation of the film response as a function of the absorbed dose was used to determine the linearity of the film response. As suggested by Zhu et al.,^(34)^ deviation of the measured net optical density of the film from the linear approximations, with zero *y* intercept, was introduced as the nonlinearity correction factor for this film. Similarly, the linearity of XR type R film was determined by exposing 21 pieces of the film to doses ranging from 10 cGy to 400 cGy and 7 pieces each with 50‐kVp, 100‐kVp, and 150‐kVp X‐ray beams. These films were read with the GretagMacbeth D19C reflection densitometer 24 h post‐exposure. The optical densities of this film were plotted as a function of the absorbed dose to determine their nonlinearity correction factors. The linearity responses of XR type T and type R films were compared with each other as well as with the linearity of model MD‐55‐2 film.

### B.4 Time dependence of the film response

Three pieces of XR type T film were exposed to doses of 842 cGy, 1684 cGy, and 3368 cGy with a 6‐MV X‐ray beam and were scanned 1 h, 3 h, 5 h, 15 h, 21 h, 44 h, 97 h, and 314 h after the exposure. Similarly, three pieces of XR type R film were exposed to a dose of 400 cGy with 50 kVp, 100 kVp, and 150 kVp beams and read at different time periods ranging from 1 h to 336 h. The responses of these films were plotted and analyzed. Variation of the film responses as a function of time were used to determine their time dependence.

### B.5 Dose‐rate dependence

Responses of seven pieces of XR type T and type R films, exposed to the same total dose using various dose rates ranging from 16.5cGy/min to 755.9cGy/min, were evaluated to determine the dose‐rate dependence of these films. The films were irradiated to a dose of 400 cGy for type T films and 368 cGy for type R films using the Therapax HF 150T superficial unit. Different dose rates were generated by increasing the film‐to‐source distance and calibrating the beam using an NIST traceable calibrated Markus ionization chamber. The exposed films were read after 24 h, and the data was analyzed in order to determine the dose‐rate dependence of these film types.

### B.6 UV light sensitivity

The effect of UV light on the XR type T and type R films was measured by exposing them to the light emitted by fluorescent tubes. One piece of each type of film was placed at a distance of approximately 1.5 m from the light source (two 40‐W tubes). The film responses were measured prior to exposure and after 10 days, 30 days, 50 days, and 60 days. The 100‐kVp calibration curves were used to convert the net film response to absorbed dose (cGy). The measured absorbed dose (Gy) was divided by the illumination of the light bulbs (lux) for each film type and plotted as a function of time. The illumination of the fluorescent tubes was determined by using the following equation^(35)^

$$\text{lux}=\frac{\text{watt}\;(80\text{lm}/W)}{m^{2}}$$

where watt is the total power of the fluorescent light tubes, assuming a luminous efficiency ^(36)^ of 80 lm/W.

## III. RESULTS AND DISCUSSIONS

The dosimetric characteristics (i.e., sensitivity, energy dependence, linearity, time dependence, dose‐rate dependence, and UV light sensitivity) of the GAFCHROMIC® XR type T and type R films were experimentally determined in air and Solid Water™ phantom material, and the results are discussed below.

### A. Film sensitivity

The responses of the XR type T film and MD‐55‐2 model, exposed to 100‐kVp and 6‐MV X‐ray beams, are compared in Figs. 1(a) and 1(b), respectively. These results indicate that for a dose of 928 cGy from a 100‐kVp X‐ray beam, the XR‐type T film is approximately 14 times more sensitive than the MD‐55‐2 film. However, for the 6‐MV beam, the sensitivity of the XR type T film is approximately (within ±10%) the same as the MD‐55‐2 film for doses lower than 1000 cGy, but the differences increase at larger doses.

**Figure 1(a):**
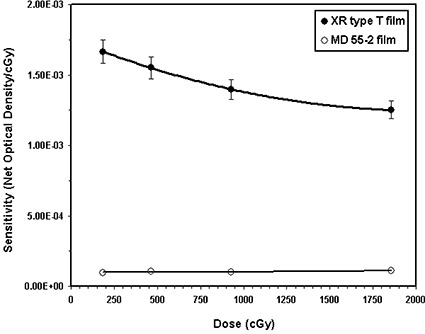
Comparison of XR type T (solid symbols) and MD‐55‐2 (open symbols) film sensitivities (optical density/cGy) as a function of dose for the 100‐kVp X‐ray beam. The solid lines are a second‐order polynomial fit through the data points.

Similarly, Fig. 1(c) compares the responses of the XR type R film and model MD‐55‐2 films exposed to a 50‐kVp X‐ray beam and measured in the cyan color spectrum. This figure indicates that for the 920 cGy absorbed dose from the 50‐kVp X‐ray beam, the XR type R film is approximately five times more sensitive than the MD‐55‐2 model. Table 2 shows the ratio of sensitivity of XR type R film to that of MD‐55‐2 film for the four different color spectra of the densitometer as a function of absorbed dose. This table indicates that for the 50‐kVp X‐ray beam the XR type R film is approximately 6.5±0.97 times more sensitive than MD‐55‐2 film.

**Table 2:** Ratio of sensitivity of XR type R film to that of MD‐55‐2 film as a function of absorbed dose for the 50‐kVp X‐ray beam, measured for the four different color spectra of the densitometer

Dose (cGy)	Black	Cyan	Magenta	Yellow
184	7.82	7.31	6.57	5.70
460	7.58	6.57	7.18	6.83
920	6.86	5.42	7.09	7.46
1840	5.54	4.12	6.03	6.17

### B. Energy dependence

Energy dependence of the XR type T and MD‐55‐2 films was measured for the energies ranging from 20 kVp to 18 MV. Figure 2(a) shows that the net optical densities of these films, normalized to the value at 6 MV, are nearly constant (within ±10%) for the photon energies greater than 300 keV. For energies less than 300 keV, the MD‐55‐2 film shows a decrease in optical density with decreasing beam energy. However, the relative optical density of XR type T films increases and then decreases as energy decreases, with a maximum value at 100 kVp. The MD‐55‐2 film results are consistent with the published data.^(^ ^8^ ^,^ ^25^ ^,^ ^27^ ^,^ ^37^ ^,^ ^38^ ^)^ This figure indicates that the response of the XR type T film at 41 keV (i.e., 100 kVp) is approximately 7.5 times larger than that of 6‐MV photons. However, the variation of the MD‐55‐2 film response in the energy range of 41 keV to 6 MV was found to be negligible (less than 5%). Table 3(a) shows relative optical densities of the XR type T film as a function of the effective beam energy.

**Figure 1(b):**
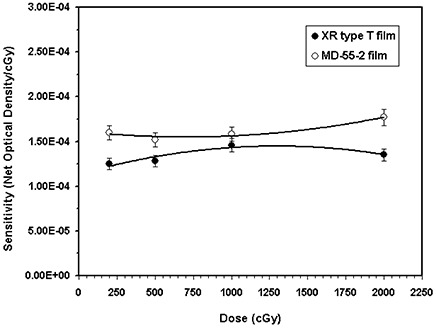
Comparison of XR type T (solid symbols) and MD‐55‐2 (open symbols) film sensitivities (optical density/cGy) as a function of dose for the 6‐MV X‐ray beam. The solid lines are a second‐order polynomial fit through the data points.

**Table 3(a):** The relative optical densities of the XR type T film as a function of beam energy ranging from 11 keV to 6000 keV, normalized to the 6 MV (i.e., 2000 keV) X‐ray beam

Effective energy (keV)	Normalized optical density
11	1.66
16	3.48
18	3.52
19	4.13
25	5.30
30	6.27
41	7.45
50	6.85
79	4.70
140	1.96
380	0.85
2000	1.00
6000	0.93

Similarly, the energy dependence of XR type R film was measured for the different energies ranging from 20 kVp to 18 MV and compared with those of the MD‐55‐2 film. Figure 2(b) shows the relative optical density of the XR type R and MD‐55‐2 films for the cyan color spectrum of the densitometer as a function of effective beam energy. This figure indicates that the relative response of both MD‐55‐2 and XR type R films, for energies greater than 650 keV, is nearly constant (within ±10%). For energies less than 650 keV, the relative optical density of XR type R films increases and then decreases as energy decreases, with a maximum value at 100 kVp, whereas the optical density of MD‐55‐2 decreases monotonically with decreasing beam energy. The MD‐55‐2 film results are consistent with the published data.^(^ ^8^ ^,^ ^27^ ^,^ ^37^ ^,^ ^38^ ^)^ Figure 2(b) indicates that at 41 keV (i.e., 100 kVp) the response of the XR type R film is approximately six times larger than that of 6‐MV photons while the MD‐55‐2 film, measured with the same technique, showed approximately 30% smaller response than a 6‐MV beam. Figure 2(c) shows the net optical density of the XR type R film as a function of beam energy and color of the densitometer. This figure indicates that the absolute value of the responses is largest for the cyan color spectrum, which is the manufacturer's recommended spectrum for use of fluoroscopic skin dose measurements. Table 3(b) shows relative optical densities of the XR type R film as a function of the effective beam energy.

### C. Linearity of the film response

Figure 3(a) shows the measured optical density of XR type T films as a function of absorbed dose in the range 10 cGy to 3368 cGy for 100‐kVp and 6‐MV X‐ray beams. Although both curves appear more linear at lower optical densities, they show some nonlinearity as the optical density increases. The solid lines show the second‐order polynomial fit through the measured data points. The dashed lines with open symbols in the figure represent the nonlinearity correction factor calculated as a ratio of the measured optical density and linear approximation with no *y*‐intercept, as suggested by Zhu et al.^(34)^ With a second‐order polynomial fit, the agreement between the fitted data and measured data is within 5% throughout the range, except at the lowest dose of 46 cGy, where 35% disagreement has been observed. Conversely, a linear fit shows a poorer agreement across the entire range: within 10% for doses above 500 cGy, and degenerating to an approximate 40% difference at 93 cGy for 100‐kVp X‐ray beam. The film uncertainty was obtained by measuring the film response at different segments of the film and comparing that with the average value. For high‐energy beams, the statistical fluctuation of the film response was found to be less than ±5% for doses greater than 800 cGy, and ranged from ±22% at 200 cGy to ±1.6% at 3200 cGy. However, for low‐energy beams the statistical fluctuation was less than ±5% for doses above 50 cGy.

**Figure 1(c):**
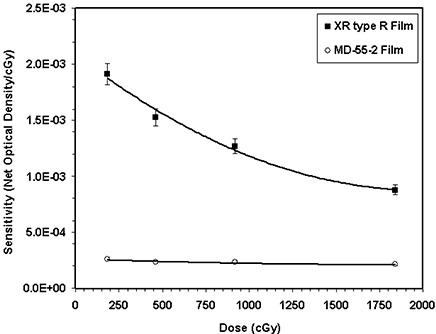
Comparison of XR type R (solid symbol) and MD‐55‐2 (open symbols) film sensitivities (optical density/cGy) for the cyan color spectrum of the densitometer as a function of dose for the 50‐kVp X‐ray beam. The solid lines are a second‐order polynomial fit through the data points.

**Figure 2(a):**
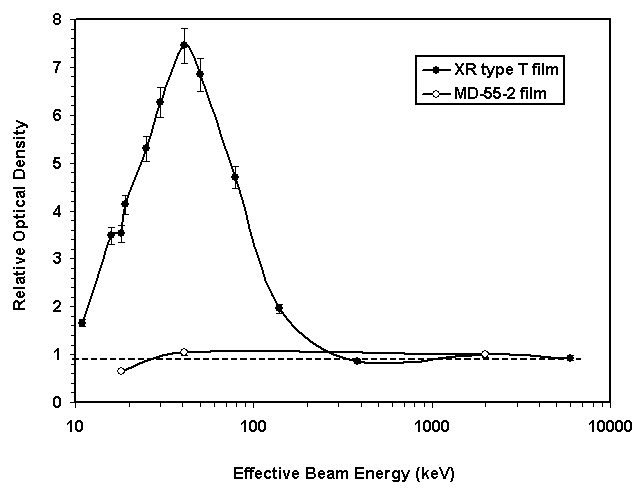
Energy dependence of XR type T and MD‐55‐2 film responses shown as the variation of the relative optical density versus the effective beam energy. These films were exposed to a fixed total dose of 800 cGy, with different effective beam energies ranging from 11 keV to 6000 keV. The solid lines connect the data points.

**Figure 2(b):**
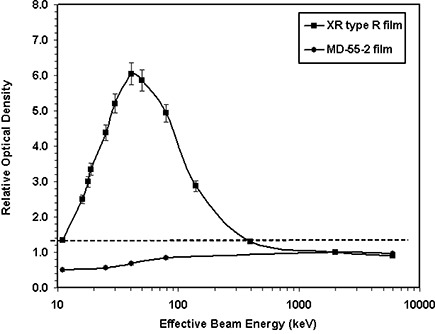
Energy dependence of XR type R and MD‐55‐2 film responses, for the cyan color of the densitometer, shown as the variation of the relative optical density versus the effective beam energy. These films were exposed to a total fixed dose of 400 cGy with different effective beam energies ranging from 11 keV to 6000 keV. The solid lines connect the data points.

**Figure 2(c):**
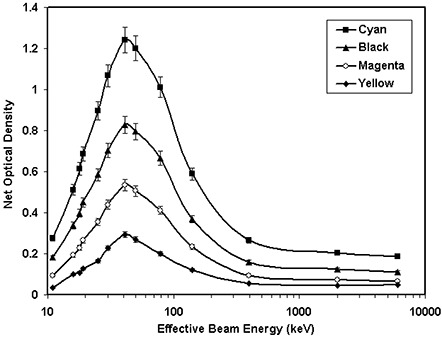
The net optical density of XR type R film as a function of effective beam energy for the cyan, black, magenta, and yellow colors of the densitometer. These films were exposed to a total fixed dose of 400 cGy with different effective beam energies ranging from 11 keV to 6000 keV. The solid lines connect the data points.

The response of XR type R films as a function of absorbed dose, ranging from 10 cGy to 400 cGy for 50‐kVp, 100‐kVp, and 150‐kVp X‐ray beams for the cyan color of the densitometer, is plotted in Fig. 3(b). Also, the response of this film to a 6‐MV X‐ray beam for doses in the range of 10 cGy to 3368 cGy for the cyan color of the densitometer is shown in Fig. 3(c). These figures indicate that the type R film response is nonlinear for both the low‐energy and high‐energy beams. With a second‐order polynomial fit, the agreement between measured and fitted data for low‐energy X‐ray beams was found to be within 10% for doses above 10 cGy. However, at 10 cGy the fitted curve disagrees with the measured data by approximately 128%. Similarly, with the polynomial fit, the 6‐MV X‐ray beam showed an agreement within 9% for doses more than 50 cGy and degenerated to 140% at 10 cGy. Conversely, a linear fit to the measured data showed a poorer agreement across the entire range. Tables 4(a) and 4(b) show the nonlinearity correction factor calculated as a ratio of the measured optical density to linear approximation, with no *y*‐intercept, for four different colors of the densitometer.^(34)^ No significant difference in percentage deviation from linearity was observed for 50‐kVp, 100‐kVp, and 150‐kVp X‐ray beams; their average values for the four colors of densitometer are shown in Table 4(a). These results indicate a nonlinearity correction factor greater than 10% for low‐energy X‐ray beams with doses less than 200 cGy and high‐energy X‐ray beams with doses less than 1700 cGy. For high‐energy beams, the statistical uncertainty of this film was found to be less than ±5.6% for doses greater than 200 cGy, and ranged from 15% at 25 cGy to 2% at 3200 cGy. However, for low‐energy beams this uncertainty was found to be less than ±5% for doses above 25 cGy.

**Table 3(b):** The relative optical density of XR type R film as a function of beam energy ranging from 11 keV to 6000 keV for four different color spectra of the densitometer, normalized to the 6‐MV (i.e., 2000 keV) X‐ray beam

		Normalized optical density		
Effective energy (keV)	Black	Cyan	Magenta	Yellow
11	1.46	1.35	1.31	0.73
16	2.70	2.50	2.73	2.20
18	3.18	3.00	3.23	2.40
19	3.61	3.35	3.72	2.82
25	4.68	4.38	4.99	3.64
30	5.62	5.21	6.18	5.07
41	6.62	6.05	7.54	6.51
50	6.36	5.86	7.13	5.96
79	5.33	4.93	5.80	4.47
140	2.95	2.87	3.28	2.68
380	1.28	1.30	1.31	1.24
2000	1.00	1.00	1.00	1.00
6000	0.89	0.90	0.92	1.04

**Table 4(a):** Nonlinearity correction factors for XR type R film in the dose range 10 cGy to 400 cGy for low‐energy X‐ray beams for the four colors of densitometer. Since no significant differences were observed between the 50‐kVp, 100‐kVp, and 150‐kVp X‐ray beams, an average value of the three energies is shown.

		Nonlinearity correction factors (low‐energy photons)		
Dose (cGy)	Cyan	Black	Magenta	Yellow
10	1.2	0.9	0.8	0.0
25	1.4	1.3	0.9	0.0
50	1.5	1.4	1.2	0.9
100	1.3	1.3	1.2	1.1
200	1.1	1.1	1.1	1.0
300	1.0	1.0	1.0	1.0
400	0.9	0.9	1.0	1.0

**Table 4(b):** Nonlinearity correction factors for XR type R film in the dose range 11 cGy to 3368 cGy for the 6‐MV X‐ray beam, for different color spectra of the densitometer

		Nonlinearity correction factor (high‐energy photons)		
Dose (cGy)	Cyan	Black	Magenta	Yellow
11	1.7	0.9	0.0	0.0
26	1.5	2.3	2.2	5.7
53	1.5	1.9	1.7	4.5
105	1.7	1.8	1.5	2.1
211	1.6	1.6	1.3	0.5
421	1.5	1.5	1.4	1.2
842	1.3	1.3	1.2	1.1
1684	1.1	1.1	1.1	1.0
3368	0.9	0.9	1.0	0.9

**Figure 3(a):**
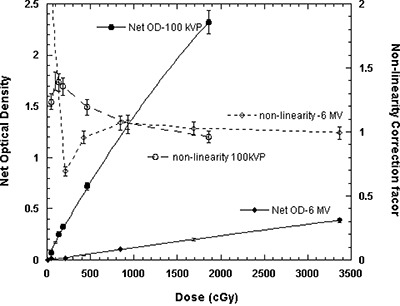
The net optical density and nonlinearity correction factors for XR type T films as a function of absorbed dose to water for 100‐kVp and 6‐MV X‐ray beams. The solid lines are a second‐order polynomial fit through the measured optical densities. The dashed lines connect the calculated nonlinearity correction points.

**Figure 3(b):**
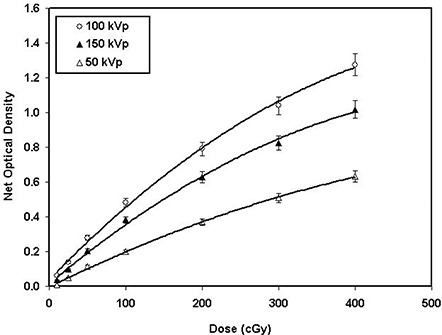
The net optical density of XR type R film as a function of absorbed dose to water for 50‐kVp (open triangles), 100‐kVp (open circles), and 150‐kVp (closed triangles) X‐ray beams for the cyan color spectrum of the densitometer. The solid lines are a second‐order polynomial fit through the data points.

**Figure 3(c):**
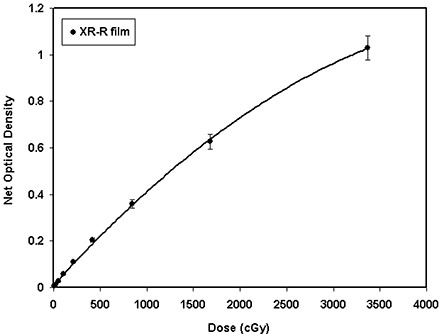
The net optical density of XR type R film for the cyan color spectrum of the densitometer as a function of absorbed dose to water for the 6‐MV X‐ray beam. The solid line is a second‐order polynomial fit through the data points.

### D. Time dependence

Optical densities of the GAFCHROMIC® films as a function of time after the exposure were used to evaluate their time dependence. Figure 4(a) shows the post‐irradiation film response for the XR type T films for the absorbed doses of 842 cGy, 1684 cGy, and 3368 cGy. This figure indicates that the film response for a given dose changes by approximately 16% within the first 24 h after exposure and an additional 4% for the next 24 h. However, no significant changes (less than 2%) have been observed up to 314 h after exposure.

**Figure 4(a):**
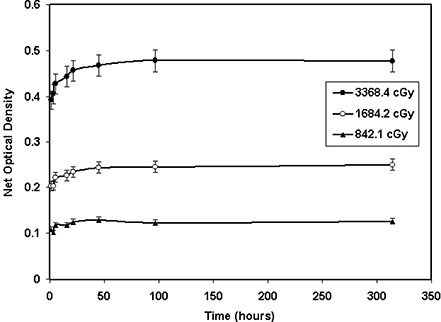
Variation of net optical densities of XR type T film as a function of time after exposure for doses of 3368 cGy, 1684 cGy, and 842 cGy. The solid lines connect the data points.

Similarly, Fig. 4(b) shows the post‐irradiation film response of the cyan color spectrum of the XR type R film for a fixed total dose of 400 cGy with 50‐kVp, 100‐kVp, and 150‐kVp X‐ray beams. An increase of approximately 16% in optical densities was noted between the first hour and 24th hour of exposure, and a growth of approximately 4% for the next 24 h and approximately 2% for the following 300 h. A similar trend was observed for the other color spectra of the densitometer. Tables 5(a) and 5(b) provide detailed information regarding the time dependence of both XR type T and type R films (cyan color), respectively.

**Table 5(a):** Post‐exposure density growth of XR type T films as a function of time and absorbed dose, relative to the value at 1 h after exposure

Time (h)	842.1 cGy	Relative film response 1684.2 cGy	3368.4 cGy
1.0	1.00	1.00	1.00
3.3	1.00	1.00	1.03
5.4	1.09	1.09	1.09
15.5	1.09	1.11	1.13
21.3	1.16	1.15	1.16
44.6	1.20	1.20	1.19
97.0	1.20	1.21	1.22
314.5	1.20	1.23	1.22

**Table 5(b):** Post‐exposure density growth of XR type R films as a function of time and absorbed dose, relative to the value at 1 h after exposure, for the cyan color of the densitometer

Time (h)	50 kVp	Relative film response 100 kVp	150 kVp
1	1.00	1.00	1.00
12	1.18	1.13	1.13
24	1.15	1.14	1.14
48	1.21	1.18	1.19
168	1.29	1.20	1.22
336	1.27	1.22	1.25

**Figure 4(b):**
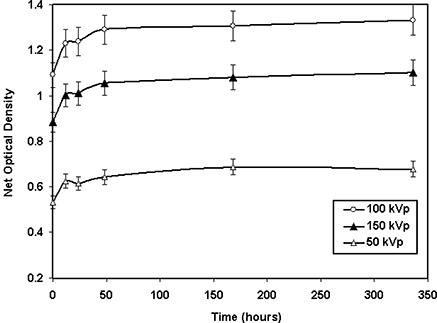
Variation of net optical densities (cyan color) of XR type R film as a function of time after exposure for a dose of 400 cGy using a 50‐kVp, 100‐kVp, and 150‐kVp X‐ray beam. The solid lines connect the data points.

### E. Dose‐rate dependence

To accommodate a wide range of clinical applications using the radiochromic film, a detailed knowledge of the film's dose‐rate dependence is required. Dose‐rate dependence of the XR type T radiochromic films for a given total dose was measured using different dose rates in the range 16.5cGy/min to 755.9cGy/min. Figure 5(a) shows the variation of the film response as a function of dose rate. The solid line in this figure represents a linear fit to the measured data. As seen in this figure, the approximately zero slope of this line indicates no dose‐rate dependence of this film, within an experimental uncertainty of ±5%. Similarly, the responses of XR type R films to different dose rates ranging from 30 cGy/min to 699 cGy/min, with a total dose of 368 cGy, are shown in Fig. 5(b). This figure shows a random variation of the film response around a linear fit with approximately zero slope, to within ±5%, indicating that the film is dose‐rate‐independent.

**Figure 5(a):**
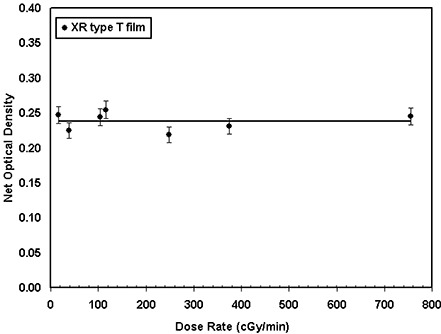
Variation of the optical density of the XR type T film as a function of dose rate ranging from 16 cGy/min to 756 cGy/min. The solid line is a linear fit through the data points.

**Figure 5(b):**
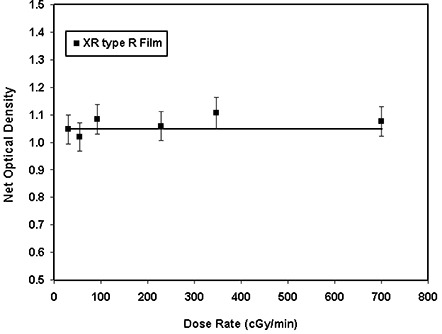
Variation of the optical density (cyan color) of the XR type R film as a function of dose rate ranging from 30 cGy/min to 700 cGy/min. The solid line is a linear fit through the data points.

### F. UV light sensitivity

The sensitivities of the GAFCHROMIC® XR type T and type R films to UV light were measured to determine the change in the film optical density due to room light during radiation dosimetry. The impact of room light (i.e., fluorescent tubes) was measured and converted to equivalent absorbed dose using a calibration curve from a 100‐kVp X‐ray beam. Figure 6(a) shows the variation of the equivalent absorbed dose per unit illuminance (lux) after 10, 31, 52, and 60 days of exposure to the room light for the XR type T film. The dose received by this film for 10 days of continuous exposure to two 40‐W tubes at a distance of 1.5 m was measured to be approximately 6.74 cGy. Figure 6(b) shows the variation of equivalent absorbed dose after 3, 8, 15, and 30 days of exposure to room light for the XR type R film. These results indicate that a short exposure of these films to room light during the measurements will not have a significant impact on the radiation dosimetry. A one‐day exposure to room light at a distance of 1 m or a 4‐day exposure at a distance of 2 m may add a dose of 0.8 cGy to both XR type T and type R films, which is insignificant (less than 3%) for measuring doses greater than 25 cGy.

**Figure 6(a):**
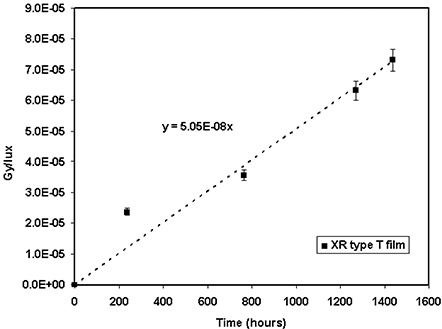
Ultraviolet light dose contamination per lux at a distance of 1 m for dosimetry with XR type T film. The solid line represents a linear fit through the measured data points.

**Figure 6(b):**
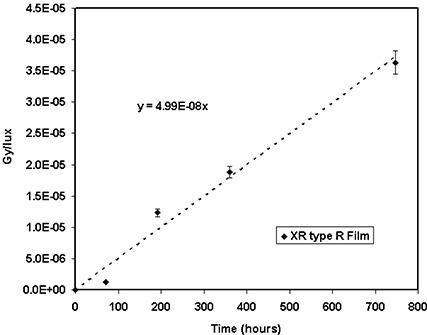
Ultraviolet light dose contamination per lux at a distance of 1 m for dosimetry with XR type R film read with the cyan color of the densitometer. The solid line represents a linear fit through the measured data points.

## IV. CONCLUSION

The dosimetric characteristics of both XR type T and type R films were determined experimentally for both low‐ and high‐energy X‐ray beams. The color of XR type R and T films changes from amber to brownish‐green and orange to blackish‐green, respectively, depending on the level of exposure. The results of these two film types were compared with each other as well as with model MD‐55‐2 film. These results indicated that type T films were approximately 14 times more sensitive than MD‐55‐2 film, for 928 cGy from a 100‐kVp beam. However, for high‐energy beams their sensitivities were found to be within ±10%. Table 2 shows that for a dose of 920 cGy from a 50‐kVp beam, the XR type R film is approximately 5.42 times more sensitive than MD‐55 film for the cyan color of the densitometer. Also, this result indicated that the average ratio of sensitivities of type R to MD‐55 film, for all four colors of the densitometer, was found to be 6.5±0.97. In addition to the sensitivity, the type T and R films have a lower threshold than the MD‐55‐2 film (100 cGy for type T, 25 cGy for type R and 200 cGy for MD‐55‐2 film, reported in this investigation). Therefore, type T and R films are superior to MD‐55‐2 film for the measurements of low absorbed doses in the diagnostic energy range.

Energy dependence of XR type T and R film response was measured and compared with that of MD‐55‐2 film. As shown in Figs. 2(a) and 2(b), the optical density of MD‐55‐2 film is nearly constant for high‐energy photons. However, at lower energies the optical density of this film decreases with decreasing energy. At 18 keV, the optical density of MD‐55‐2 film is approximately 40% smaller than that of a 6‐MV beam. These results are consistent with the published data.^(^ ^8^ ^,^ ^25^ ^,^ ^27^ ^,^ ^37^ ^,^ ^38^ ^)^ In addition, Figs. 2(a) and 2(b) show that the variation of measured optical density of XR type T and type R films, for photon beams greater than 300 keV and 650 keV, respectively, is within ±10%. However, for lower energies the relative optical densities of XR type T and R films increase to a value of approximately 7 at 41 keV (i.e., 100 kVp) and then decrease to a value of approximately 1.5 at 11 keV (20 kVp). Therefore, for high‐energy beams, the energy dependence of the new films is similar to that of MD‐55‐2, but at low energies new films present a larger variation of optical density compared with MD‐55‐2 film.

The results of these investigations show that the responses of XR type R and T films as a function of absorbed dose can be approximated better with a second‐order polynomial than with a linear function. The deviation from linearity for these films was introduced as a nonlinearity correction factor, similar to that of MD‐55 film as suggested by Zhu et al.^(34)^ As shown in Fig. 3(a), the nonlinearity corrections for the XR type T film are within 10% for doses above 500 cGy and degenerate to approximately 40% at approximately 93 cGy. Table 4(a) shows the nonlinearity correction factors for type R film for the low‐energy photons as a function of the color of the densitometer. These results indicate that for cyan, the nonlinearity corrections of the type R film are in the range 1.5 to 0.9 for doses ranging from 10 cGy to 400 cGy. However, for high‐energy photons, the nonlinearity corrections for cyan are in the range 1.7 to 0.9 for the doses ranging from 11 cGy to 3368 cGy, as shown in Table 4(b). The nonlinearity corrections for high‐energy beams were found to be highest for the yellow color of the densitometer. The statistical fluctuation of XR type T film response, for high‐energy beams, was found to be less than ±5% for doses greater than 800 cGy, while for low‐energy beams, the fluctuation was less than ±5% for doses above 50 cGy. For XR type R film, the statistical fluctuation was found to be less than ±5.6% for doses greater than 200 cGy, while for low‐energy beams, the fluctuation was within ±5% for doses above 25 cGy.

The optical densities of XR type T and R films were found to increase by approximately 16% within the first 24 h after exposure and an additional 4% for the next 24 h. No significant (less than 2%) changes were found approximately 360 h after the exposure. These results are consistent with the data obtained for XR type films by Giles and Murphy^(39)^ and ISP.^(40)^ In addition, Ali et al.^(41)^ and Meigooni et al.^(33)^ have shown that the optical density of MD‐55 films increases by approximately 16% after 24 h from the time of exposure. Therefore, no time dependence correction is needed if the patient and calibration films are exposed and read simultaneously. However, if the patient film is read immediately after exposure, but the calibration is read 24 h after exposure, the optical density of the patient film must be increased by approximately 16%. Responses of both XR type T and type R films were found to be dose‐rate‐independent. The results of these investigations showed a variation of ±5% for the dose rates ranging from 16 cGy/min to 755 cGy/min. Saylor et al.^(9)^ showed a ±5% variation in optical densities of HD‐810 film for dose rates ranging from 2 cGy/min to 20 000 cGy/min. In addition, Niroomand‐Rad et al.^(27)^ state that MD‐55 film response is independent of dose rates in the clinical range of 200 cGy/min to 400 cGy/min. More recently, Giles and Murphy^(39)^ and ISP^(40)^ showed that XR type films are dose‐rate‐independent within ±5%. These published results support the findings of our investigation.

Several investigators have noted that GAFCHROMIC^®^ films are sensitive to UV light and hence suggest that proper care and handling can eliminate UV contamination.^(^ ^27^ ^,^ ^32^ ^,^ ^33^ ^,^ ^40^ ^)^ The results of our investigation show an insignificant effect of UV light on the sensitivities of GAFCHROMIC^®^ XR type T and type R films. These results indicate that the dose received by XR type T and type R films from a continuous exposure to two 40‐W tubes for a period of 4 days, at a distance of 2.0 m, is equivalent to approximately 0.8 cGy dose from a 100 kVp X‐ray beam. This amount of UV contamination is insignificant (less than 3%) for clinical applications, which require a dose greater than 25 cGy.

In summary, the higher sensitivity and lower threshold of the XR type R and type T films, at low photon energies, is superior to other film models. This can be helpful for radiation dosimetry, particularly for low‐dose radiation measurements during interventional radiological procedures. However, the higher energy dependence of the new films relative to the MD‐55‐2 films should be taken into consideration for absolute dose measurements. For the new films, use of a previously calibrated film‐strip without a clear identification of the beam quality may introduce an erroneous result. Therefore, the new film types should be either calibrated with the same beam quality as the measured beam or corrected for the differences in the film response using the data in Figure 2. No significant differences in linearity, time dependence, and dose‐rate dependence of the new films to that of MD‐55‐2 films have been observed. Because of the opaque backing material on the XR type R films, they must be read with a reflective densitometer, while the XR type T film can either be read with a laser scanner or a white light scanner with appropriate filtration.

## ACKNOWLEDGMENT

The authors would like to thank Dr. David Lewis from ISP for providing the GAFCHROMIC^®^ films used in these investigations, as well as Dr. Walter Miller for providing the Tc^99m^ for this project. The authors note their appreciation for Mr. Shahid B. Awan, Mr. Curtis Baker, Mr. Venkata Rachabatthula, and Mrs. Jenny Clark, for their valuable editorial comments and suggestions during the preparation of this manuscript.

## Supporting information

Supplementary Material

Supplementary Material

Supplementary Material

Supplementary Material

Supplementary Material
