# Supplementary figures and images for: Dosimetric evaluation of GAFCHROMIC® XR type T and XR type R films
Source: J Appl Clin Med Phys. 2005 Mar 17;6(1):114–34. doi: 10.1120/jacmp.v6i1.2051 (PMC5723503; doi:10.1120/jacmp.v6i1.2051)

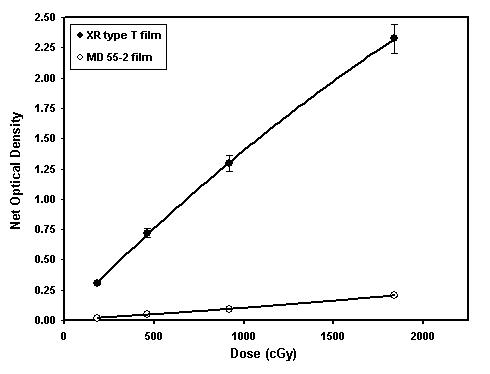

Supplement: Supplementary file 1 — Supplementary Material [file ACM2-6-114-s001.jpg]

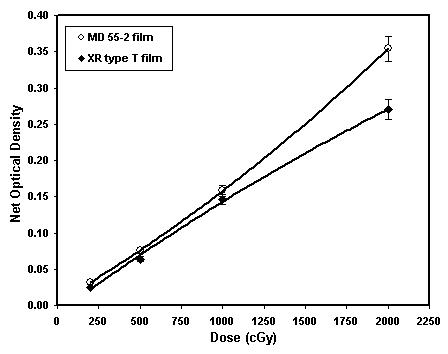

Supplement: Supplementary file 2 — Supplementary Material [file ACM2-6-114-s002.jpg]

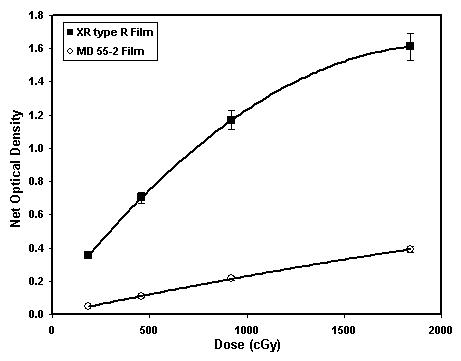

Supplement: Supplementary file 3 — Supplementary Material [file ACM2-6-114-s003.jpg]

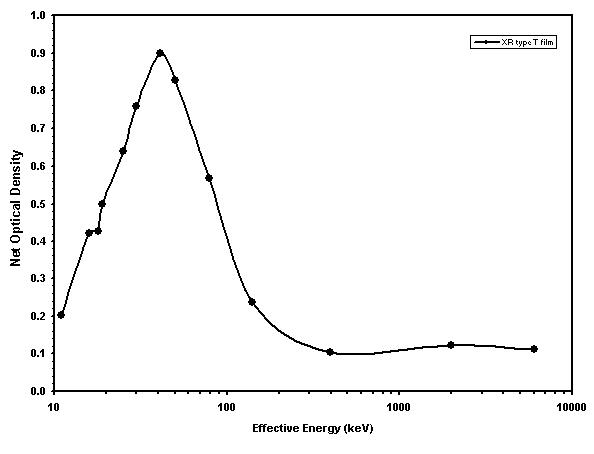

Supplement: Supplementary file 4 — Supplementary Material [file ACM2-6-114-s004.jpg]

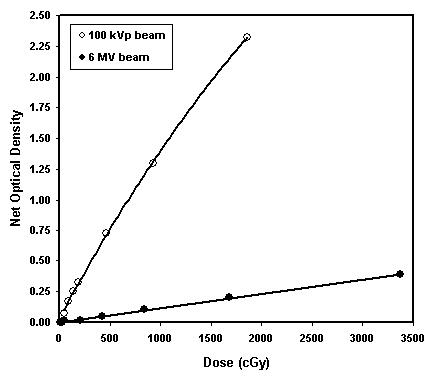

Supplement: Supplementary file 5 — Supplementary Material [file ACM2-6-114-s005.jpg]
